# Insights Into Long Non-Coding RNA and mRNA Expression in the Jejunum of Lambs Challenged With *Escherichia coli* F17

**DOI:** 10.3389/fvets.2022.819917

**Published:** 2022-04-12

**Authors:** Weihao Chen, Xiaoyang Lv, Weibo Zhang, Tingyan Hu, Xiukai Cao, Ziming Ren, Tesfaye Getachew, Joram M. Mwacharo, Aynalem Haile, Wei Sun

**Affiliations:** ^1^College of Animal Science and Technology, Yangzhou University, Yangzhou, China; ^2^International Centre for Agricultural Research in the Dry Areas, Addis Ababa, Ethiopia; ^3^Joint International Research Laboratory of Agriculture and Agri-Product Safety of Ministry of Education of China, Yangzhou University, Yangzhou, China

**Keywords:** lamb, *E. coli* F17, lncRNA, mRNA, RNA-Seq, machine learning

## Abstract

It has long been recognized that enterotoxigenic *Escherichia coli* (ETEC) is the major pathogen responsible for vomiting and diarrhea. *E. coli* F17, a main subtype of ETEC, is characterized by high morbidity and mortality in young livestock. However, the transcriptomic basis underlying *E. coli* F17 infection has not been fully understood. In the present study, RNA sequencing was conducted to explore the expression profiles of mRNAs and long non-coding RNAs (lncRNAs) in the jejunum of lambs who were identified as resistant or sensitive to *E. coli* F17 that was obtained in a challenge experiment. A total of 772 differentially expressed (DE) mRNAs and 190 DE lncRNAs were detected between the *E. coli* F17—resistance and *E. coli* F17-sensitive lambs (i.e., *TFF2, LOC105606142, OLFM4, LYPD8, REG4, APOA4*, TCONS_00223467, and TCONS_00241897). Then, a two-step machine learning approach (RX) combination Random Forest and Extreme Gradient Boosting were performed, which identified 16 mRNAs and 17 lncRNAs as potential biomarkers, within which *PPP2R3A* and TCONS_00182693 were prioritized as key biomarkers involved in *E. coli* F17 infection. Furthermore, functional enrichment analysis showed that peroxisome proliferator-activated receptor (PPAR) pathway was significantly enriched in response to *E. coli* F17 infection. Our finding will help to improve the knowledge of the mechanisms underlying *E. coli* F17 infection and may provide novel targets for future treatment of E. coli F17 infection.

## Introduction

Diarrhea is the most commonly reported disease associated with infection by a complex mixture of bacteria in young animals. Among them, *Escherichia coli* (*E. coli*) is the major pathogenic bacterium responsible for diarrhea (1). Pathogenic *E. coli* have been divided into five pathotypes based on the virulence properties and clinical signs of the host: enterotoxigenic *E. coli* (ETEC), enterohemorrhagic *E. coli* (EHEC), enteropathogenic *E. coli* (EPEC), enteroinvasive *E. coli* (EIEC), and diffusely enteroadherent *E. coli* [DAEC, (2)].

Among these pathotypes, ETEC has been identified as the major agent of *E. coli*-related diarrhea (3–6). Mechanistically, ETEC adheres to intestinal epithelial cells (IECs), leading to the production and replication of enterotoxins (7). Clinical reports revealed that ETEC infection exhibits enteropathogenicity and enterotoxigenicity, causing increased mortality and clinical signs such as severe vomiting and diarrhea (8). The fimbrial adhesins, F5 (9), F17 (10), F18 (11), and F41 (12) are associated with ETEC, mainly in young animals. *E. coli* F17, one of the main subtypes of ETEC, has been reported as the major pathogen associated with ETEC-related diarrhea worldwide and is responsible for high morbidity and mortality (13–15). The growing prevalence of *E. coli* F17 has renewed the sense of urgency for *E. coli* F17 research.

Over 100,000 long non-coding RNAs (lncRNAs) have now been identified, and although the roles of most of them are still unknown, lncRNAs have been shown to play key roles in gene regulation and cellular functions (16). Increasing evidence has shown that lncRNAs contribute to immune activity at multiple levels during *E. coli* infection. For example, lncRNA-TUB was shown to mediate *E. coli*-induced inflammatory factor secretion and *Staphylococcus aureus* adhesion to epithelial cells (17), and lncRNA-XIST was found to mediate *E. col*-induced inflammatory response in bovine mastitis (18). These data indicate that how specific lncRNAs can regulate *E. coli* infection. However, remarkably few comparative studies of the roles of lncRNAs and mRNAs in *E. coli* infections, especially *E. coli* F17, have been conducted.

For this study, lambs that were resistant or sensitive to *E. coli* F17 were obtained in a challenge experiment. RNA sequencing (RNA-Seq) was performed to obtain the transcriptomic profiles. Then, differential expression analysis, machine learning analysis, integrative network, and functional enrichment analyses were performed for a deep insight into lncRNA and mRNA in response to *E. coli* F17 infection. Our results will help to improve the knowledge of the mechanisms underlying *E. coli* F17 infection and may provide novel targets for future treatment of *E. coli* F17 infection.

## Materials and Methods

### Ethics Approval

All the lamb experimental procedures used in the study were reviewed and approved by the Experimental Animal Welfare and Ethical of Institute of Animal Science, Yangzhou University (No: NFNC2020-NFY-6), and were performed in accordance with the Regulations for the Administration of Affairs Concerning Experimental Animals approved by the State Council of the People's Republic of China.

### Sample Collection

All experimental lambs were supplied by the Xilaiyuan Agriculture Co., Ltd. (Jiangsu Providence, China). *E. coli* F17-resistant and *E. coli* F17-sensitive lambs were detected from a challenge experiment of *E. coli* F17 (DN1401, fimbrial structural subunit: F17b, fimbrial adhesin subunit: Subfamily II adhesins, originally isolated from diarrheic calves) as described in our previous report (19).

Briefly, 50 healthy newborn lambs were randomly selected and reared on lamb milk replacer free of antimicrobial additives and free of probiotics from when they were 1 day old to 3 days old. At 3 days after birth, lambs were divided into high-dose and low-dose challenge groups. Lambs in the high-dose and low-dose challenge groups were orally gavaged with 50.0 and 1.0 ml of actively growing culture of *E. coli* F17 (1 × 10^9^ CFU/ ml) for 4 days, respectively. Then, 10 healthy lambs in the high-dose challenge group (antagonism candidate group) and 10 lambs with severe diarrhea in low-dose challenge group (sensitive candidate group, evaluate *via* stool consistency scoring) were euthanized by administering pentobarbital overdose. Histopathological examination and bacteria plate counting of the intestinal contents were conducted to evaluate the severity of the diarrhea. Results showed that severe pathological intestinal tissues were observed in *E. coli* F17-sensitive candidate lambs, while relatively healthy intestinal tissue were observed in *E. coli* F17-antagonism candidate lambs. Intestinal contents bacteria plate counting demonstrated that bacteria in the intestinal contents of *E. coli* F17-sensitive candidate lambs (1.22^*^10^9^ on average) were significantly higher than that of *E. coli* F17-antagonism candidate lambs (3.37^*^10^7^ on average). Detailed results of intestinal histopathological detection and bacterial counting can be found in in our previous report (19).

Finally, six healthy lambs with mild intestinal pathology in the high-dose challenge group (antagonism group, AN) and six lambs with severe diarrhea in the low-dose challenge group (sensitive group, SE) with severe intestinal pathology were selected. Proximal jejunum tissue was collected and snap-frozen in liquid nitrogen for RNA isolation.

### RNA Extraction and Sequencing

Ribonucleic acid was extracted from the jejunum tissue using TRIzol^®^ per the manufacturer's instructions (Invitrogen, Carlsbad, CA, USA). The quality of the extracted RNA was determined using an RNA Nano 6000 Assay Kit, and RNA integrity number (RIN) was obtained using an Agilent 2100 Bioanalyzer with RIN ≥ 8.0 as the threshold.

The mRNA and lncRNA libraries were constructed using a NEB Next^®^ Ultra™ RNA Library Prep Kit for Illumina^®^ per the manufacturer's instructions (NEB, Ipswich, MA, USA). The RNA libraries were sequenced on an Illumina HiSeq^TM^ 2500 platform with PE150 strategy (paired-end 150 bp) by Beijing Novogene Technology Co., Ltd (Beijing, China).

### Sequencing Data Analysis

The raw reads were obtained in FASTQ format. Low-quality reads, namely, reads with adapters, reads that contained N (wherein the proportion of unidentified bases > 0.2%), and low-quality reads (quality scores < Q20; i.e., bases with sQ ≤ 5 more than 50% of all reads) were removed. Clean reads were generated and then mapped to the *Ovis aries* reference genome (Oar_v4.0) using Hisat2 (20). StringTie (21) was used to assemble the mRNA transcripts. Then, coding and non-coding RNA candidates from the transcripts were distinguished using Coding-Non-Coding-Index [CNCI, (22)], Coded Potential Calculator-2 [CPC2, (23)], and Pfam-scan [PFAM, (24)] software. Non-coding RNA candidates with lengths > 200 nt, and with exon numbers ≥ 2 were identified as candidate lncRNAs.

The expected number of Fragments Per Kilobase of transcript sequence per Million fragments sequenced [FPKM, (25)] was used to estimate the expression levels of candidate lncRNA and mRNA transcripts. Differentially expressed (DE) lncRNAs and DE mRNAs were identified between AN and SE groups using edgeR R library (26). lncRNAs and mRNAs were considered significantly DE as the threshold of padj (*p*-values adjusted by Benjamini and Hochberg's approach) < 0.01.

### Identification of mRNA/lncRNA Biomarkers Using Machine Learning Method

To identify potential lncRNA and mRNA biomarkers for *E. coli* F17 infection, one two-step machine learning approach (RX) combination Random Forest [RF, (27)] and Extreme Gradient Boosting [XGBoost, (28)] were performed. The randomForest library and XGBoost library in R software was applied for the analysis. The detailed strategy for RX were described in our previous research (29).

Briefly, we systematically examined a range of parameters (Ntree and mtry values for RF, colsample and eta for XGBoost), and out-of-bag (OOB) error rate was used for determining the derive minimum hyperparameter values required for final analysis. For biomarkers identification, firstly, RF was applied to select the subset of lncRNAs and mRNAs with positive values of variable important measures (VIMs). Then, these selected lncRNAs and mRNAs from RF were further assessed by XGBoost. Similarly, XGBoost produces a VIM rank for the genes named “Gain.” In the current study, the VIM value of individual variable (mRNA and lncRNA) denotes the relative contribution of the variable for each tree in the model. The higher the “Gain” value, the more important the variable is for generating a classification between lambs AN and SE lambs. Hence, variable with a high “Gain” were therefore prioritized as potential mRNA/lncRNA biomarkers for *E. coli* F17 infection.

### Integrative Network Analysis

To elucidate the interaction between the mRNAs and lncRNAs, cis- and trans-target genes of lncRNA were predicted. Coding genes located 100 kb upstream or downstream of the corresponding lncRNAs were considered cis-target genes. To identify candidate trans-target genes, Pearson correlation coefficients were calculated between the expression level of coding genes and corresponding lncRNAs. Coding genes were considered trans-target genes for |correlation| ≥ 0.95.

Based on the target gene prediction, the interactions between DE lncRNAs and DE mRNAs were used to construct the lncRNA-mRNA integrative network using Cytoscape v3.7.2 software (30).

### Functional Analysis

Gene Ontology (GO) and Kyoto Encyclopedia of Genes and Genomes (KEGG) enrichment analyses were performed for the DE mRNAs and target genes of the DE lncRNAs GOseq R library (31) and KO-Based Annotation System (KOBAS) programs (32), followed by a Fisher's exact test with a false discovery rate (FDR) multiple test correction to assess the statistical significance (*p* < 0.05).

### Real-Time QPCR

To validate the RNA-Seq data, five mRNAs and five lncRNAs were randomly selected. The house-keeping gene GAPDH was selected as the reference gene, and the primers were designed using Primer Premier 5 software. The sequences of the selected mRNAs and lncRNAs were shown in Supplementary Table S1.

Total RNA was extracted from the jejunum tissue of 12 lambs (6 AN and 6 SE) processed for sequencing using TRIzol^®^ per the manufacturer's instructions (Invitrogen, Carlsbad, CA, USA). The first strand of cDNA was prepared using FastKing gDNA Dispelling RT per the manufacturer's instructions (Vazyme Biotech, Nanjing, Jiangsu, China).

The PCR thermocycler program procedure was as follows: 37°C for 15 min, followed by 85°C for 5 s. The reaction mixture contained 4.0 μl 5× FastKing-RT SuperMix 2.0 μl, 2.0 μg Total RNA, and RNase-free ddH_2_O to a total volume to 20 μl. The quality of the cDNA was evaluated by housekeeping gene amplification and stored at −20°C until use.

Real-time qPCR was performed in triplicate with cDNA to validate the reliability of RNA-Seq data following the SYBR Green I method with 1 cycle at 95°C for 15 min, followed by 40 cycles at 95°C for 10 s, and 60°C for 30 s. The dissociation curve was analyzed after amplification.

The 2^−ΔΔCt^ method (33) was used to calculate expression level of selected lncRNAs and mRNAs. The results were shown as relative expression level (log_2_FoldChange mean ± standard error) using GraphPad Prism 6 software.

## Results

### Global RNA-Seq Data

The average numbers of raw reads were 85,523,999 (AN) and 84,450,970 (SE); the average numbers of clean reads were 84,384,636 (AN), and 83,112,267 (SE); and the average mapping rates for the AN and SE were 98.67 and 98.41%, respectively. Detailed characteristics of the two libraries are shown in Table 1.

**Table 1:** Summary of the sequencing data.

**Sample name**	**Raw reads**	**Clean reads**	**Clean bases**	**Error rate (%)**	**Q20 (%)**	**Q30 (%)**	**GC content (%)**
AN1	86,448,964	85,310,470	12.97G	0.03	97.55	93.27	51.32
AN2	82,985,976	82,314,372	12.45G	0.03	97.00	91.76	46.32
AN3	81,095,934	79,701,960	12.16G	0.03	97.49	93.16	51.61
AN4	94,502,330	93,722,960	14.18G	0.03	97.25	92.49	48.07
AN5	84,496,940	83,246,004	12.67G	0.03	97.45	93.06	50.25
AN6	83,613,850	82,012,052	12.54G	0.03	97.49	93.19	54.43
SE1	82,325,980	81,420,394	12.21G	0.03	97.31	92.67	52.18
SE2	83,101,628	81,439,640	12.22G	0.03	97.39	93.00	48.07
SE3	83,731,304	82,241,834	12.34G	0.03	97.45	93.09	49.79
SE4	80,794,124	79,478,658	11.92G	0.03	96.90	91.99	56.07
SE5	92,174,900	90,902,860	13.64G	0.03	97.35	92.99	49.55
SE6	84,577,884	83,190,218	12.48G	0.03	96.54	91.14	49.89

*AN and SE represent antagonism group and sensitive group, respectively. Error rate % represent average error rate of sequencing of the single base*.

**Figure 1:**
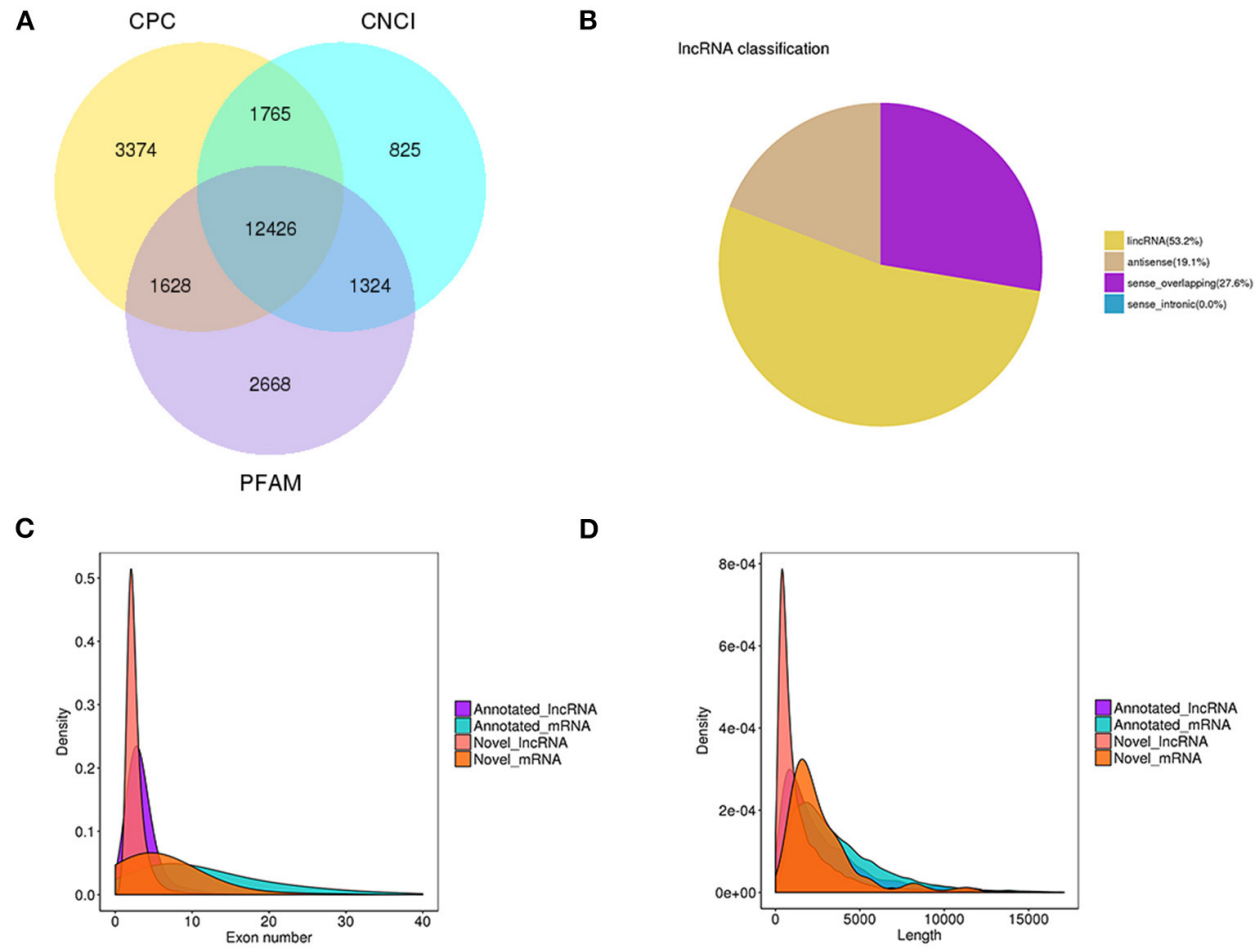
lncRNA filter/classification and exon/length distribution of identified lncRNAs and mRNAs. **(A)** Filter of identified lncRNAs. **(B)** Classification of identified lncRNAs. **(C)** Exon number distribution of identified lncRNAs and mRNAs. **(D)** Length distribution of identified lncRNAs and mRNAs.

We identified a total of 20,601 mRNAs and 12,426 lncRNAs, of which 9,148 of the lncRNAs were novel and 3,278 were annotated lncRNAs (Figure 1A). Among the novel lncRNAs, 53.2, 27.6, and 19.1% were identified as lncRNAs, sense-overlapping lncRNAs, and antisense lncRNAs, respectively (Figure 1B). The 20,601 mRNAs and 12,426 lncRNAs were screened for in-depth analyses.

The mRNAs had an average of 5.76 exons, whereas most of the lncRNAs had an average of 2.71 exons (Figure 1C). Most of the mRNAs were 500-3,000 bp long with an average length of 2285.65 bp, whereas most of the lncRNAs were 200-1,000 nt long with an average length of 1713.33 nt (Figure 1D). The detailed information of mRNAs and lncRNAs can be found in Supplementary Table S2.

### Differentially Expressed mRNAs and lncRNAs

**Figure 2:**
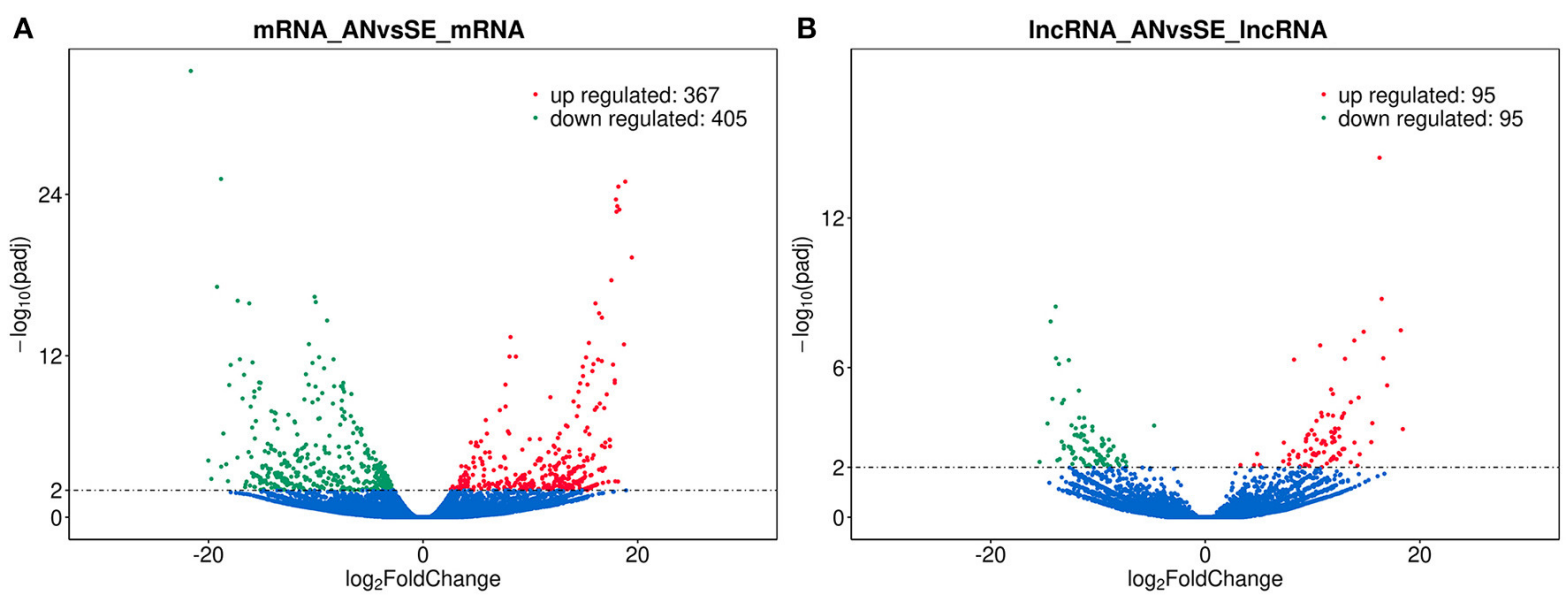
Volcano plot of differentially expressed (DE) mRNAs **(A)** and lncRNAs **(B)** in antagonism group (AN) vs. sensitive group (SE), where red and green represent up or downregulation, respectively.

We identified 772 DE mRNAs between the AN and SE libraries, within which 367 were upregulated and 405 downregulated (Figure 2A). One hundred ninety DE lncRNAs were identified between the AN and SE libraries, within which 95 were upregulated and 95 were downregulated (Figure 2B). Details are provided in Supplementary Table S3.

**Figure 3:**
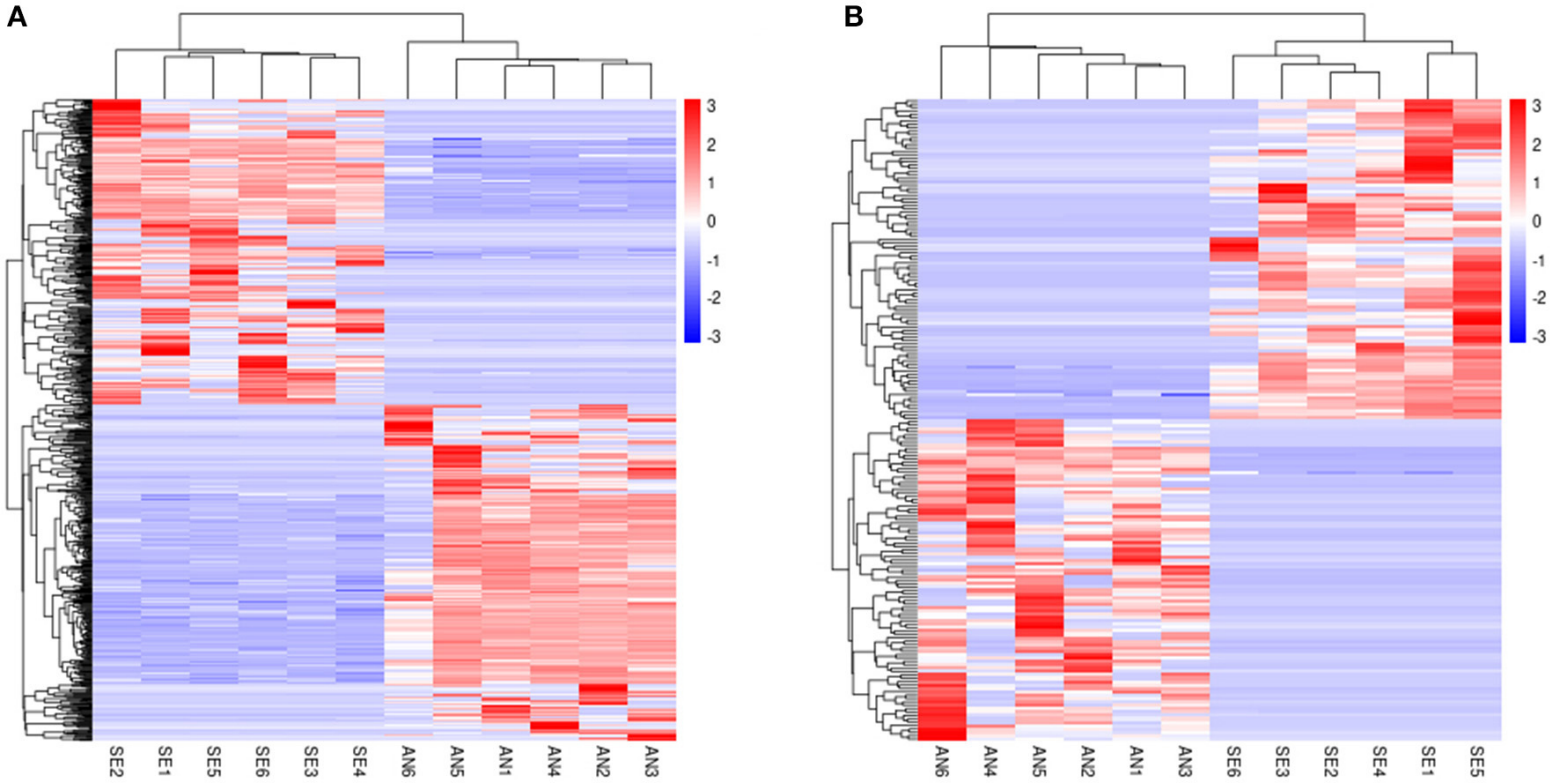
Heat map of DE mRNAs **(A)** and DE lncRNAs **(B)**.

Cluster analysis was performed and heat maps of the DE lncRNAs (Figure 3A) and DE mRNAs (Figure 3B) revealed a clear different expression pattern clearly between AN and SE.

### Identification of Potential mRNA and lncRNA Biomarkers for *E. Coli* F17 Infection

The final parameters used for RF and XGBoost analyses of mRNA and lncRNA datasets were chosen based on a systematic evaluation of a range of values. Details can be seen in Supplementary Table S3.

**Figure 4:**
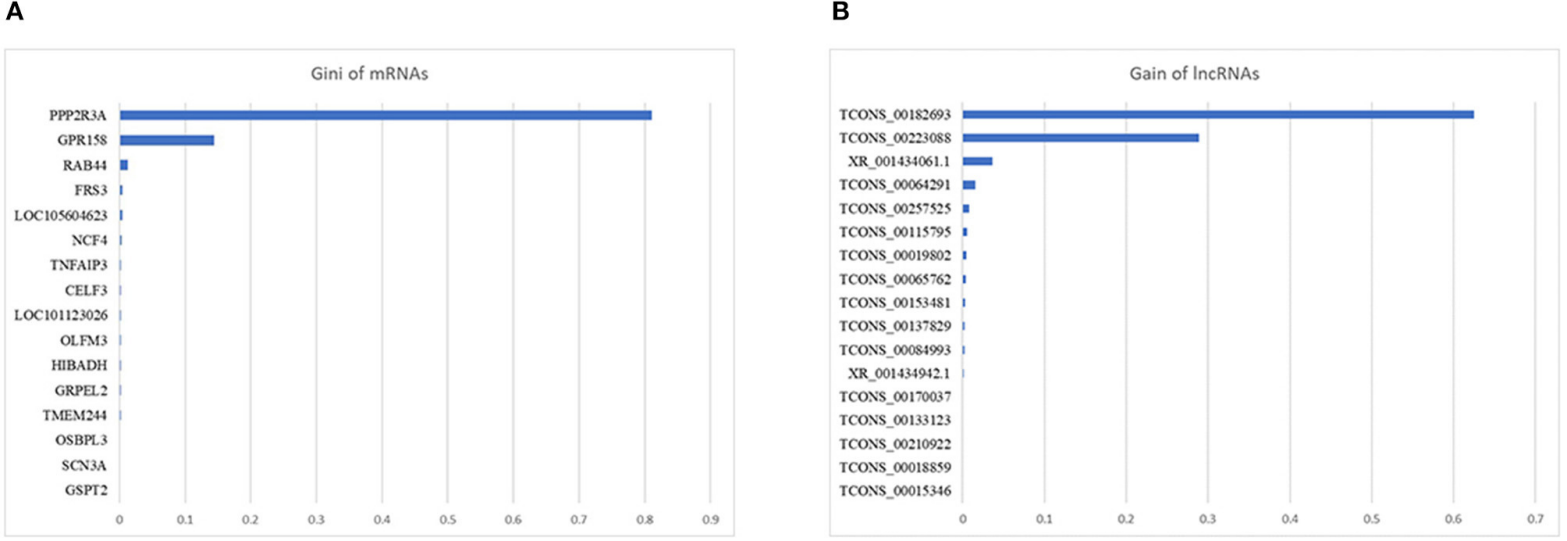
Gain value of the mRNAs **(A)** and lncRNAs **(B)** selected by the two-step machine learning approach (RX).

For mRNA biomarkers identification, 4,424 mRNAs with positive VIM value were identified by RF and 16 mRNAs were further selected by XGBoost, of which the top three mRNAs with highest Gain value were *PPP2R3A* (0.81), *GPR158* (0.14), and *RAB44* (0.01). For lncRNA biomarkers identification, 2,700 lncRNAs with positive VIM value were identified by RF and 17 lncRNAs were further selected by XGBoost, of which the top three lncRNAs with highest Gain value were TCONS_00182693 (0.63), TCONS_00223088 (0.29), and XR_001434061.1 (0.04). Figure 4 illustrates the mRNAs (Figure 4A) and lncRNAs (Figure 4B) selected by RX. The detailed results of RX can be found in Supplementary Table S4.

### Target Gene Prediction and Integrative Network Analysis

Overall, 15,379 cis-target genes of 12,481 corresponding lncRNAs and 5,756 trans-target genes of 5,171 corresponding lncRNAs were predicted. Detailed prediction results are provided in Supplementary Tables S5, S6. Based on the DE analysis, 48 DE lncRNAs were found to cis-regulate 38 DE mRNAs, and 115 DE lncRNAs were found to trans-regulate 264 DE mRNAs. A total of 950 DE lncRNA-mRNA pairs were used for the subsequent integrative network analysis.

The connection number of each candidate node in the integrative network was calculated. The top three most connected DE lncRNAs were TCONS_00133120 (61), TCONS_00070741 (36), and TCONS_00009486 (36); and the top three most connected DE mRNAs were *CES3* (33), *SLC5A12* (28), and *SOAT2* (20). The interaction network is in shown in Supplementary Figure S1 and detailed information is provided in Supplementary Table S7.

### GO and KEGG Enrichment Analysis

To explore the mechanisms underlying *E. coli* F17 infection, GO and KEGG enrichment analysis were conducted on the up and downregulated DE mRNAs and DE lncRNAs identified in AN vs. SE.

**Figure 5:**
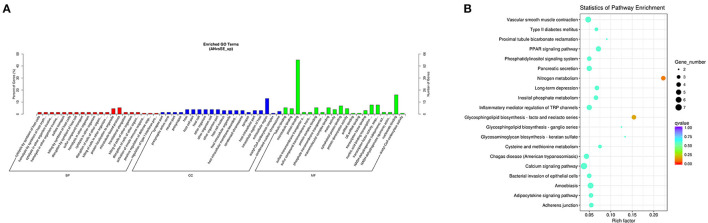
Top annotated gene ontology (GO) terms **(A)** and Kyoto Encyclopedia of Genes and Genomes (KEGG) pathways **(B)** of upregulated DE mRNAs.

The upregulated DE mRNAs were significantly enriched in 159 GO terms and 14 KEGG pathways. The top enriched GO terms Figure 5A were multi-organism cellular process (GO:0001071), extracellular region (GO:0005576), and binding (GO:0005488) under the biological process (BP), cellular component (CC), and molecular function (MF), respectively. The top enriched KEGG pathways Figure 5B were nitrogen metabolism (oas00910), amoebiasis (oas05146) and peroxisome proliferator-activated receptor (PPAR) signaling pathway (oas03320).

**Figure 6:**
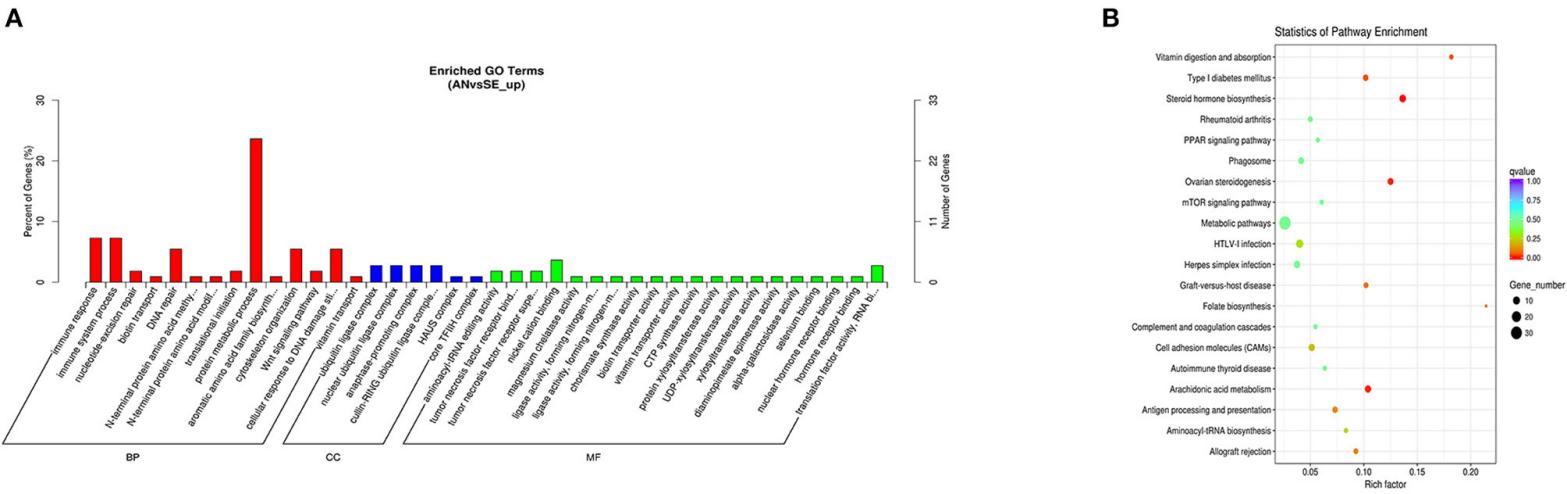
Top annotated GO terms **(A)** and KEGG pathways **(B)** of cis-target genes of upregulated DE lncRNAs.

The cis-target genes of upregulated DE lncRNAs were significantly enriched in 47 GO terms and 18 KEGG pathways. The top enriched GO terms Figure 6A were regulation of protein metabolic process (GO:0019538), ubiquitin ligase complex (GO:0000151), and nickel cation binding (GO:0016151) under the BP, CC, and MF categories, respectively. The top enriched KEGG pathways Figure 6B were metabolic pathways (oas01100), HTLV-I infection (oas05166), and steroid hormone biosynthesis (oas00140).

**Figure 7:**
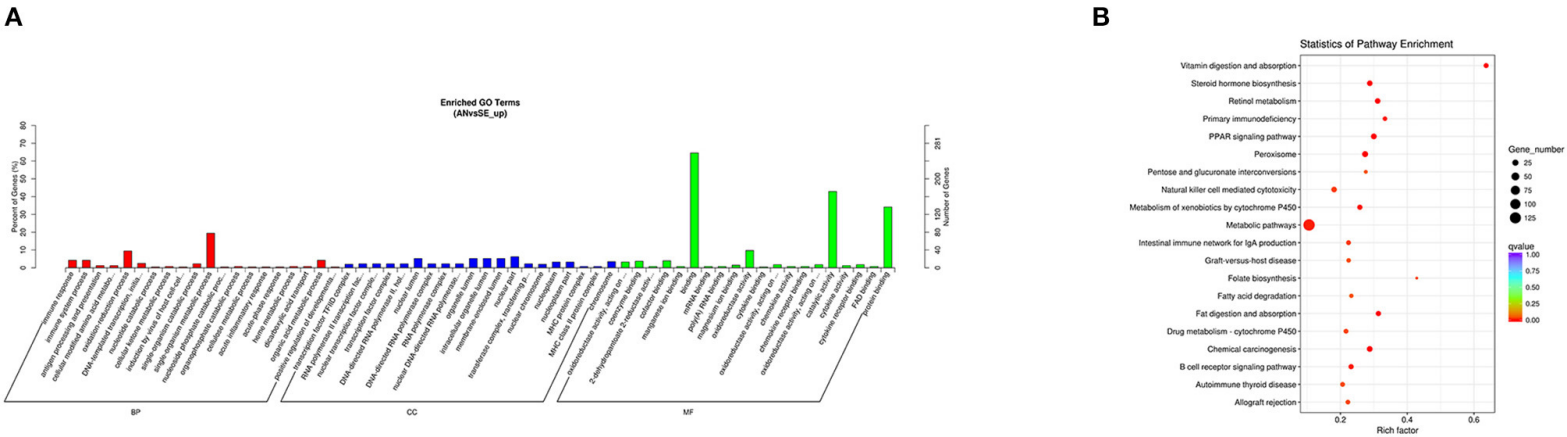
Top annotated GO terms **(A)** and KEGG pathways **(B)** of trans-target genes of upregulated DE lncRNAs.

The trans-target genes of the upregulated DE lncRNAs were significantly enriched in 103 GO terms and 49 KEGG pathways. The top enriched GO terms Figure 7A were single-organism metabolic process (GO:0044710), nuclear part (GO:0044428), and binding (GO:0005488) under the BP, CC, and MF categories, respectively. The top enriched KEGG pathways Figure 7B were metabolic pathways (oas01100), peroxisome (oas04146) and PPAR signaling pathway (oas03320).

**Figure 8:**
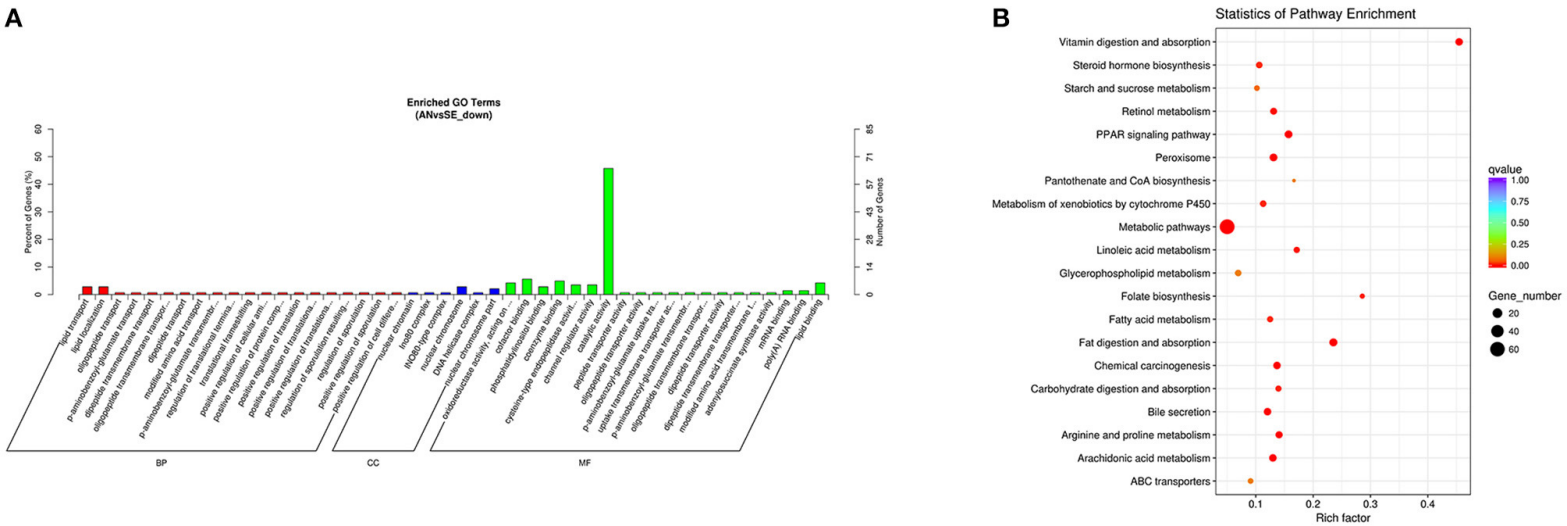
Top annotated GO terms **(A)** and KEGG pathways **(B)** of downregulated DE mRNAs.

The downregulated DE mRNAs were significantly enriched in 109 GO terms and 33 KEGG pathways. The top enriched GO terms Figure 8A were oxidation-reduction process (GO:0055114), nuclear chromosome (GO:0000228), and catalytic activity (GO:0003824) under the BP, CC, and MF categories, respectively. The top enriched KEGG pathways Figure 8B were metabolic pathways (oas01100), fat digestion and absorption (oas04975), and PPAR signaling pathway (oas03320).

**Figure 9:**
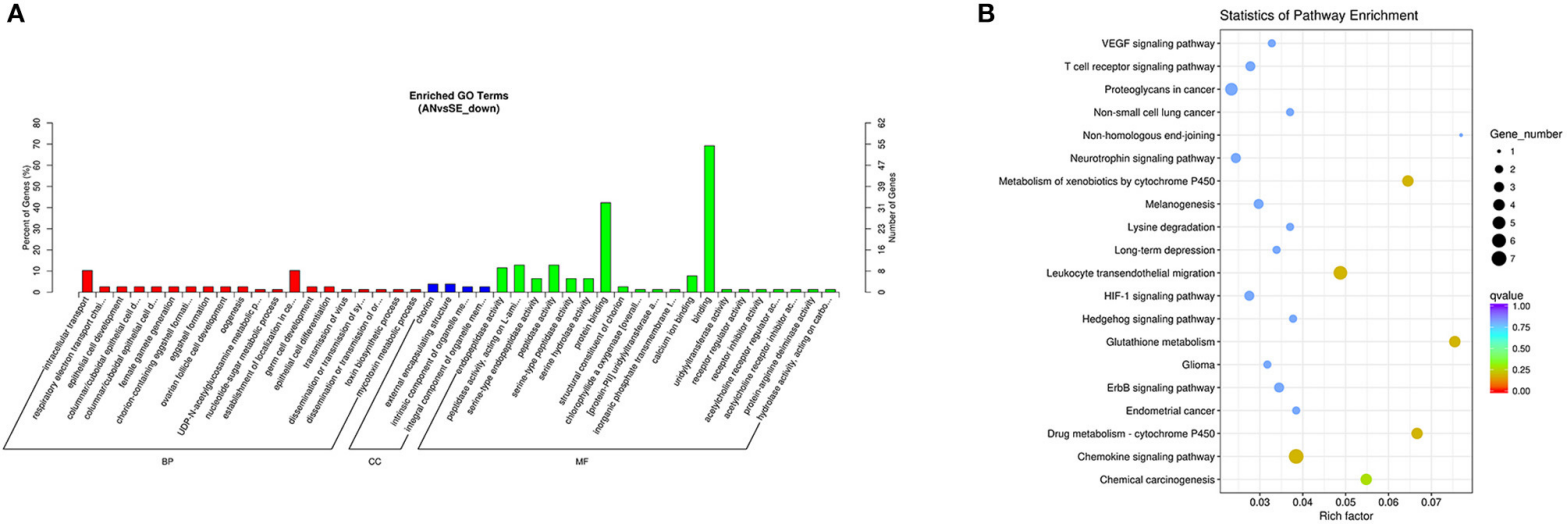
Top annotated GO terms **(A)** and KEGG pathways **(B)** of cis-target genes of the downregulated DE lncRNAs.

The cis-target genes of the downregulated DE lncRNAs were significantly enriched in 78 GO terms and 6 KEGG pathways. The top enriched GO terms Figure 9A were intracellular transport (GO:0046907), chorion (GO:0042600), and binding (GO:0005488) under the BP, CC, and MF categories, respectively. The top enriched KEGG pathways Figure 9B were chemokine signaling pathway (oas04062), leukocyte transendothelial migration (oas04670), and glutathione metabolism (oas00480).

**Figure 10:**
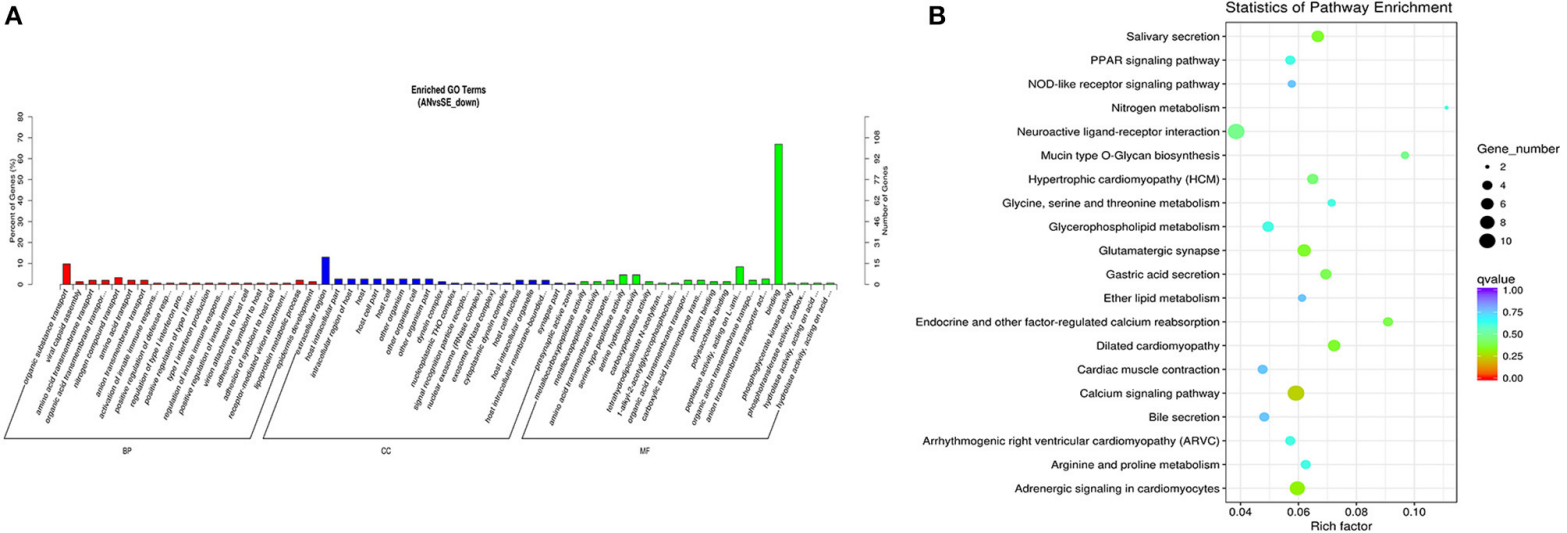
Top annotated GO terms **(A)** and KEGG pathways **(B)** of trans-target genes of the downregulated DE lncRNAs.

The trans-target genes of downregulated DE lncRNAs were significantly enriched in 109 GO terms and 12 KEGG pathways. The top enriched GO terms Figure 10A were biological adhesion (GO:0022610), extracellular region (GO:0005576), and binding (GO:0005488) under the BP, CC, and MF categories, respectively. The top enriched KEGG pathways Figure 10B were neuroactive ligand-receptor interaction (oas04080), calcium signaling pathway (oas04020), and adrenergic signaling in cardiomyocytes (oas04261).

Detailed results of GO and KEGG enrichment analysis are provided in Supplementary Tables S8, S9.

### Validation of the RNA-Seq Data

**Figure 11:**
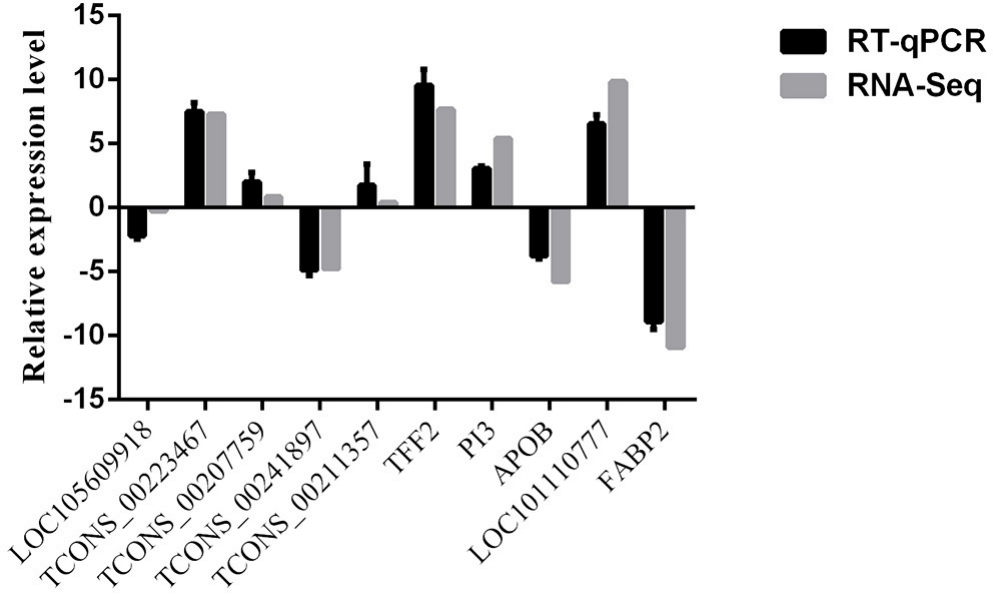
Comparisons of the results of the RNA-Seq and RT-qPCR analyses of selected lncRNAs and mRNAs in AN vs. SE.

The expression level of selected lncRNAs and mRNAs obtained by RT-qPCR were compared with those obtained by RNA-Seq are shown in Figure 11. The expression patterns of selected lncRNAs and mRNAs were similar between RNA-Seq and RT-qPCR, indicating the reproducibility and reliability of our sequencing data.

## Discussion

Considering the susceptibility of jejunum to *E. coli* F17 (19) and globally high prevalence of *E. coli* F17 in young livestock, we chose newborn lambs as the animal model of *E. coli* F17 infection for this study. Challenge experiments were conducted and *E. coli* F17-resistant (AN) and *E. coli* F17-sensitive (SE) lambs were identified based on histopathological examinations and bacteria plate counting of intestinal contents, and jejunum tissues were chosen for the final sequencing.

The average mapping rate of the clean reads was 98.54%, and 12,426 lncRNAs and 20,601 mRNAs were identified. According to the average FPKM values, the mRNA with the highest expression level was beta-2-microglobulin (*B2M*), a critical component of the major histocompatibility complex (MHC)-I antigen processing and presentation, which has important roles in immune control. Association was revealed between *B2M* and reaction to cancer immunotherapies in multiple organs (34, 35), however, investigations of the role of *B2M* in pathogenic *E. coli* infections are still limited. In our previous RNA-Seq research in spleen of diarrhea sheep, we found that *B2M* was an adjacent gene to DE circRNA (36). We consider that *B2M* may play various roles in the immune response to diarrhea in multiple organs. However, additional work is needed to confirm this possibility. The lncRNA with the highest expression level was TCONS_00211357, which was predicted to target several NF-κB pathway-related genes such as *TRIM38* [NF-κB activator, (37)] and *NFKBID* [NF-κB inhibitor, (38)]. Numerous studies have shown that the NF-κB pathway is involved in the host inflammatory response to *E. coli* (39–41). Our results suggest that there is a certain probability that TCONS_00211357 act as a key regulator of NF-κB pathway in immune response to *E. coli* F17 infection.

As anticipated, expression profiles of lncRNAs and mRNAs vary between the AN and SE groups. Comparisons revealed 772 DE mRNAs and 190 DE lncRNAs, of which the number of upregulated DE mRNAs and DE lncRNAs were relatively lower than that of downregulated DE mRNAs and DE lncRNAs, similar to the results reported previously for a variety *E. coli* challenged experiments (42–44). Hence, an unstable transcriptional profile may be the main reason for the serve diarrhea seen in SE lambs.

Many of the top-ranked upregulated DE mRNAs (by padj) were reported to be involved in intestinal epithelial barrier restitution, such as the trefoil factors (TFFs), *LOC105606142* (mucin-2-like), *OLFM4, REG4*, and *LYPD8*. The intestine has a double-layer physical barrier (mucus layer and IEC layer) that separates intestinal bacteria from the underlying lamina propria and deeper intestinal layers (45). Mucus, which is composed of TFFs and mucin glycoprotein (46), separates the pathogenic bacteria from direct contact with the IECs (47). The TFFs are known to be involved in mucosal restitution, protection, and proliferation, and are important stabilizers of the intestinal mucus. Here, we concentrate on two members of TFF family: *TFF2* and *TFF3*. As previously documented, *TFF3*, rather than *TFF2*, is more involved in mucosal restitution and protection, especially in intestinal immunity (48). However, in the present study, *TFF2* expression was notably higher in AN lambs than SE lambs (fold change = 204). *TFF3* was highly expressed level in all lambs, but no significant changes in its expression were detected between the AN and SE lambs. Hence, we speculated that although *TFF3* functions are important in mucosal restitution, the activation of *TFF2* may be the key antagonist for *E. coli* F17 infection. Another component of mucus, mucin glycoprotein, is formed of densely glycosylated *MUC2* mucin (49). Similar to *TFF2, LOC105606142* (mucin-2-like) expression was significantly higher in AN lambs than it was in the SE lambs. On the basis of these results, we hypothesized that these two genes, separately or together, accelerate mucosal restitution to protect the host against *E. coli* F17. Products of *E. coli* F17 (lipopolysaccharides and enterotoxin) can lead to the massive apoptosis of IECs, which forms another physical barrier below the mucus layer, inducing IECs to proliferate. In response to injury, intestinal stem cells give rise to daughter cells with the potential to proliferate to prevent IEC damage (50). In our present study, we identified 3 genes (*OLFM4, LYPD8*, and *REG4*) that regulate the immune response of IECs, and these genes were notably more highly expressed in AN lambs than they were in SE lambs. Olfactomedin 4 (*OLFM4*) is generally thought to be involved in the regulation of several important signaling pathways underlying a number of imperative cellular functions (51–53). For example, in individuals infected with *H. pylori, OLFM4* upregulation has been demonstrated as a marker of the immune response through reducing or eliminating *H. pylori* colonization (54). Given the likely cellular immune function of *OLFM4, OLFM4* upregulation in the AN lambs may suggest a potential role for *OLFM4* in the host immune response against *E. coli* F17 infection by reducing *E. coli* F17 colonization. Plaur domain-containing 8 (LYPD8) is a highly N-glycosylated glycosylphosphatidylinositol-anchored protein that is highly expressed on IECs. Recent studies reported that LYPD8 mediates segregation of pathogenic bacteria (including pathogenic *E. coli*) and epithelial cells in the intestine to preserve intestinal homeostasis (55). Considering the above evidence and our result, we infer that upregulation of *LYPD8* may contribute to intestinal defense against *E. coli* F17 by reducing attachment on IECs. However, whether *LYPD8* prevents infection with *E. coli* F17 still needs to be determined. *REG4* was found to be strongly upregulated during intestinal inflammation, and may be involved in enhancing intestinal metaplasia and growth of organoids (8). We also found that *REG4* strongly upregulated in the AN lambs, implying that *REG4* may be essential to intestinal metaplasia of *E. coli* F17-infected hosts. Taken together, our data highlight several potential mechanisms that prevent *E. coli* F17 infection in AN lambs, namely, protection from mucosal restitution (*TFF2* and *LOC105606142*) and cellular immune response (*OLFM4, LYPD8*, and *REG4*).

Based on top-ranked downregulated DE mRNAs, SE lambs had higher expression of metabolism-related genes, such as fatty acid-binding protein 2 (*FABP2)* and apolipoprotein genes (*APOA4, APOC3*, and *APOB*). *FABP2* encodes lipid chaperones that mediates multiple lipid-mediated intestinal biological function (56, 57). *FABP2* can also serve as a biomarker of intestinal inflammation, such as acute intestinal ischemia and active ulcerative colitis (58). In the present study, we found that the high expression level of *FABP2* was positively corrected with the severity of diarrhea in *E. coli* F17 hosts, indicating that it may serve as a candidate biomarker for *E. coli* F17 infection. Apolipoprotein genes has been implicated in the major functions of high-density lipoprotein, including lipid binding and dissolution, and activation of lecithin (59). Our data show that apolipoprotein genes were significantly more highly expressed in SE lambs than AN lambs, especially *APOA4* (fold change > 1,800). Given the critical roles of these genes in lipid metabolism, it is likely that the metabolic homeostasis of SE lambs was severely disrupted. To our great interest, in addition to lipid metabolism, *APOA4* also plays a role in intestinal anti-inflammatory processes, and over-expression of *APOA4* in IECs has been shown to promote differentiation and increase junctional strength (60). Thus, we speculate that in SE lambs, the proliferation and restitution of IECs were severely dysregulated, resulting in activation of *APOA4* for maintenance of junction and interaction between IECs.

Advances in lncRNAs have led to their potential in acting as gene silencers by interfering with the transcription machinery to suppress gene expression (16). The top upregulated lncRNA TCONS_00223467 was predicted to target *APOA4* and *APOC3*, two top-ranked downregulated DE mRNAs. Therefore, in the AN lambs, TCONS_00223467 may function as *APOA4/APOC3*-silencing factors, thereby maintaining the stability of IECs differentiation. The top downregulated lncRNA were TCONS_00241897. Interestingly, TCONS_00241897 was also predicted to target *LOC101110777* (WAP four-disulfide core domain protein 18-like, WFDC18-like), one of the top-ranked upregulated DE mRNAs. WFDC genes have putative roles in immunity, such as anti-HIV, anti-microbial, and cell migration activities (61). TCONS_00241897 may function as a silencer of *LOC101110777* and may be therapeutically relevant in *E. coli* F17 infection, especially the intestinal anti-inflammatory response. Taken together, our data suggests that TCONS_00223467 and TCONS_00241897 may play important roles in *E. coli* F17 infection as a gene silencer, making the prime candidates for future research.

Machine learning (ML) methods have shown promising results in identifying potential biomarkers when applied to transcriptomic datasets (62–64). In our previous study, we compared the performance of different ML methods and differential gene expression analysis methods [RF, XGBoost, RX, *t*-test, and edgeR; (29)]. Given that our previous results demonstrated that RX identified the smallest subsets of genes with the highest classification accuracy, RX was performed in the present study to identify potential mRNAs and lncRNAs biomarkers for *E. coli* F17 infection. Sixteen mRNAs and 17 lncRNAs were finally selected by RX, within which the mRNA and lncRNA with highest Gain value were *PPP2R3A* and TCONS_00182693. The protein phosphatase 2 regulatory subunit B”α (*PPP2R3A*) gene is a regulatory subunit of protein phosphatase 2A (PP2A) which regulates diverse cellular processes (65). TCONS_00182693 is the lncRNA with highest Gain value. However, not much is known about its roles in *E. coli* infection. The high Gain value of *PPP2R3A* and TCONS_00182693 demonstrated that they achieved a good performance in distinguishing AN and SE lambs in our transcriptomic datasets. Furthermore, the decision tree-based methods underlying RX (29) also indicated that certain interactivity existing between them and other mRNAs/lncRNAs was picked up by RX. Taken together, these results demonstrated that *PPP2R3A* and TCONS_00182693 may serve as reliable biomarkers for detection of *E. coli* F17 infection and reflect an important regulatory role for the phenotype under study.

The functional enrichment analyses of the DE mRNAs and target genes of the DE lncRNAs showed that immune-related terms were enriched for the upregulated DE mRNAs and lncRNAs, and that metabolic-related terms were enriched for the downregulated DE mRNAs and DE lncRNAs. Similar results have been reported previously (66, 67), further verifying our hypothesis that that the activity of immune-related genes was increased in the AN lambs and that the metabolic homeostasis was severely disrupted in the SE lambs during *E. coli* F17 infection. Notably, the PPAR signaling pathway was found enriched in KEGG enrichment analysis based on both DE lncRNAs and DE mRNAs. Mechanistically, the internalization of *E. coli* leads to the activation of intestine and liver immune system through the PPAR signaling pathway (68, 69), which support our result that the PPAR signaling pathway was linked to intestine inflammation. However, several known *E. coli* infection pathways, such as the TLR4 and NF-κB pathways, were not enriched in our study, which is inconsistent with the results of the previous studies. One potential explanation for these inconsistencies is that all experimental lambs were challenged with *E. coli* F17 in our study, while these genes were initially revealed between challenged and unchallenged individuals.

To further understand the interactions between the identified DE lncRNAs/mRNAs and the underlying intestinal immune mechanism, we constructed a DE lncRNA-mRNA integrative network that contained 950 DE lncRNA-mRNA pairs. The DE lncRNAs with the most connections were TCONS_00133120 (61), TCONS_00070741 (36), and TCONS_00009486 (36), and they were predicted to be target members of SLC family (e.g., *SLC2A5, SLC5A1*, and *SLC15A1*). The solute carrier (SLCs) family regulates the transport of molecules and have been overwhelmingly confirmed to function in cell proliferation, migration, and apoptosis (70–73). Therefore, we hypothesized that these lncRNAs may similarly regulate certain biological progress in the IECs. However, further in-depth studies are clearly needed to prove this hypothesis. The DE mRNAs with the most connections were *CES3* (33), *SLC5A12* (28), and *SOAT2* (20). Carboxylesterase 3 (*CES3*) encodes an enzyme that has a wide range of activities associated with the lipid-metabolism, has a possible preventive role in cancer (74, 75). *SLC5A12*, (*SMCT2*), and was initially reported to mediate sodium-dependent transport (76, 77), but the significance of its role in the immune response is still unclear. Sterol O-acyltransferases subtype 2 (*SOAT2*) encodes a microsomal protein and is especially expressed in intestine and liver. SOAT2 was shown to play a critical role in delaying the development of atherosclerosis (78). It is uncertain whether these top connected lncRNAs and mRNAs are sufficient to prevent *E. coli* F17 infection, but there is a high probability that they interact closely and act as key regulators of the host's response to *E. coli* F17 infection.

## Conclusion

Ribonucleic acid sequencing analysis identified 772 DE mRNAs and 190 DE lncRNAs between *E. coli* F17-resistant and *E. coli* F17-sensitive lambs. Several potential candidate mRNAs (*TFF2, LOC105606142, OLFM4, LYPD8, REG4*, and *APOA4*) and lncRNAs (TCONS_00223467 and TCONS_00241897) involved in intestinal immunity were identified. The functional enrichment analysis showed that the PPAR signaling pathway was significantly enriched in response to *E. coli* F17 infection. Together, our findings will increase the knowledge of the regulation modalities of lncRNAs and mRNAs against *E. coli* F17 infection.

## Data Availability Statement

The datasets presented in this study can be found in online repositories. The names of the repository/repositories and accession number(s) can be found at: https://www.ncbi.nlm.nih.gov/, PRJNA759095.

## Ethics Statement

The animal study was reviewed and approved by Experimental Animal Welfare and Ethical of Institute of Animal Science, Yangzhou University.

## Author Contributions

WC, XL, XC, TG, JM, AH, and WS: conceptualization. WC, XL, WZ, TH, XC, and ZR: data curation. WC, XL, and ZR: formal analysis. XC and WS: supervision. WC: writing—original draft. WC and WS: writing—review and editing. WS: funding acquisition. All authors read and approved the final manuscript.

## Funding

This work was supported by the National Natural Science Foundation of China-CGIAR (32061143036), National Natural Science Foundation of China (31872333, 32172689), Major New Varieties of Agricultural Projects in Jiangsu Province (PZCZ201739), The Projects of Domesticated Animals Platform of the Ministry of Science, Key Research and Development Plan (modern agriculture) in Jiangsu Province (BE2018354), Jiangsu Agricultural Science and Technology Innovation Fund [CX(18)2003], and Jiangsu Postgraduate Research and Innovation Program (KYCX21_3260).

## Conflict of Interest

The authors declare that the research was conducted in the absence of any commercial or financial relationships that could be construed as a potential conflict of interest.

## Publisher's Note

All claims expressed in this article are solely those of the authors and do not necessarily represent those of their affiliated organizations, or those of the publisher, the editors and the reviewers. Any product that may be evaluated in this article, or claim that may be made by its manufacturer, is not guaranteed or endorsed by the publisher.

## Supplementary Material

Supplementary Figure S1 The differentially expressed interaction network.

Supplementary Table S1 Primers and sequences of the selected mRNAs and lncRNAs for Real-time qPCR.

Supplementary Table S2 Detailed information of all mRNAs and lncRNAs.

Supplementary Table S3 Differentially expressed lncRNAs and mRNAs.

Supplementary Table S4 Details results of the two-step machine learning approach (RX).

Supplementary Table S5 Results of cis-target genes prediction of lncRNAs.

Supplementary Table S6 Results of trans-target genes prediction of lncRNAs.

Supplementary Table S7 Details of the differentially expressed (DE) interaction network.

Supplementary Table S8 Gene ontology (GO) enrichment results of differentially expressed mRNAs and target genes of differentially expressed lncRNAs.

Supplementary Table S9 Kyoto Encyclopedia of Genes and Genomes (KEGG) enrichment results of differentially expressed mRNAs and target genes of differentially expressed lncRNAs.
