# Supplementary material for: Insights Into Long Non-Coding RNA and mRNA Expression in the Jejunum of Lambs Challenged With Escherichia coli F17
Source: Front Vet Sci. 2022 Apr 12;9:819917. doi: 10.3389/fvets.2022.819917 (PMC9039264; doi:10.3389/fvets.2022.819917)
Supplement: Supplementary Figure S1 — The differentially expressed interaction network. [file Data_Sheet_1.PDF]

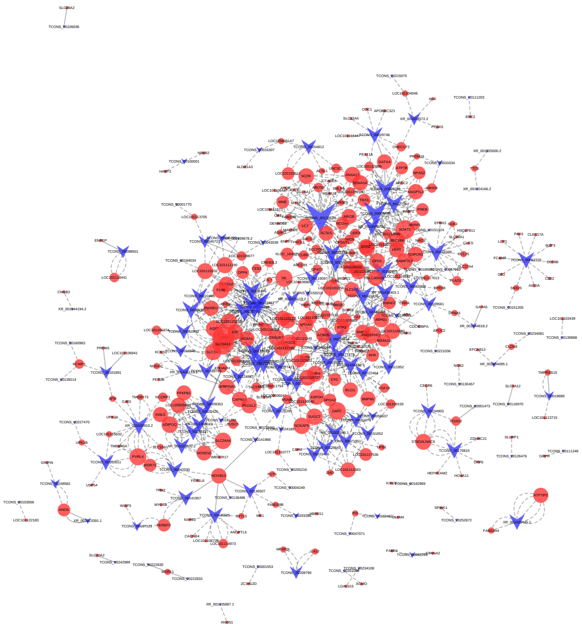

**Note:** The differentially expressed interaction network, where the “V” shape (blue) and circle (red) represent lncRNAs and mRNAs, respectively; dashed lines and solid lines represent transtarget and cis-target genes, respectively.
