# Supplementary material for: Insights Into Long Non-Coding RNA and mRNA Expression in the Jejunum of Lambs Challenged With Escherichia coli F17
Source: Front Vet Sci. 2022 Apr 12;9:819917. doi: 10.3389/fvets.2022.819917 (PMC9039264; doi:10.3389/fvets.2022.819917)
Supplement: Supplementary file 11 [file Data_Sheet_2.PDF]

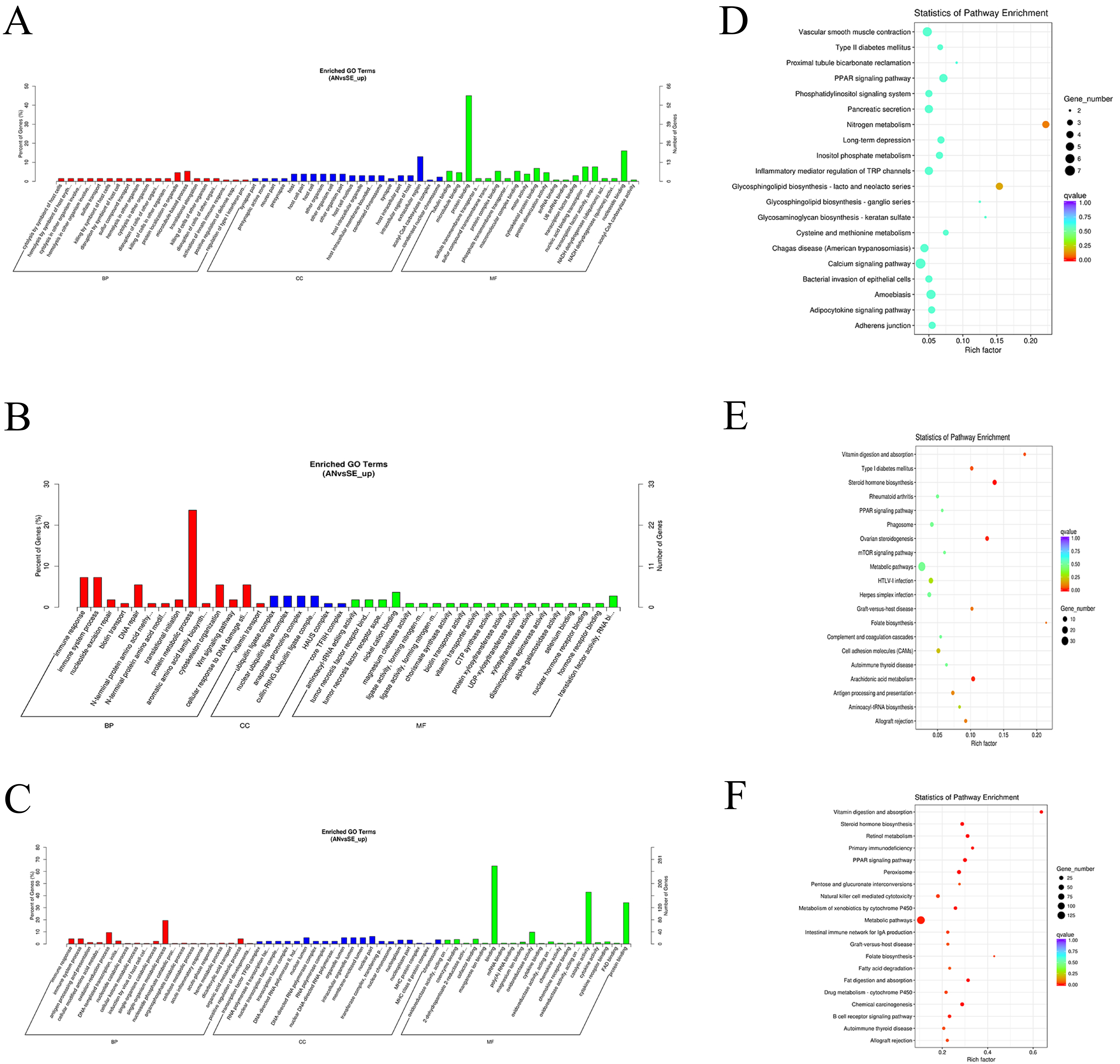

Top annotated GO terms of up-regulated DE mRNAs (A), cis-target genes (B) and trans-target genes (C) of up-regulated DE lncRNAs and top enriched KEGG pathways of up-regulated DE mRNAs (D), cis-target genes (E) and trans-target genes (F) of up-regulated DE lncRNAs.
