# Supplementary figures and images for: Insights Into Long Non-Coding RNA and mRNA Expression in the Jejunum of Lambs Challenged With Escherichia coli F17
Source: Front Vet Sci. 2022 Apr 12;9:819917. doi: 10.3389/fvets.2022.819917 (PMC9039264; doi:10.3389/fvets.2022.819917)

C

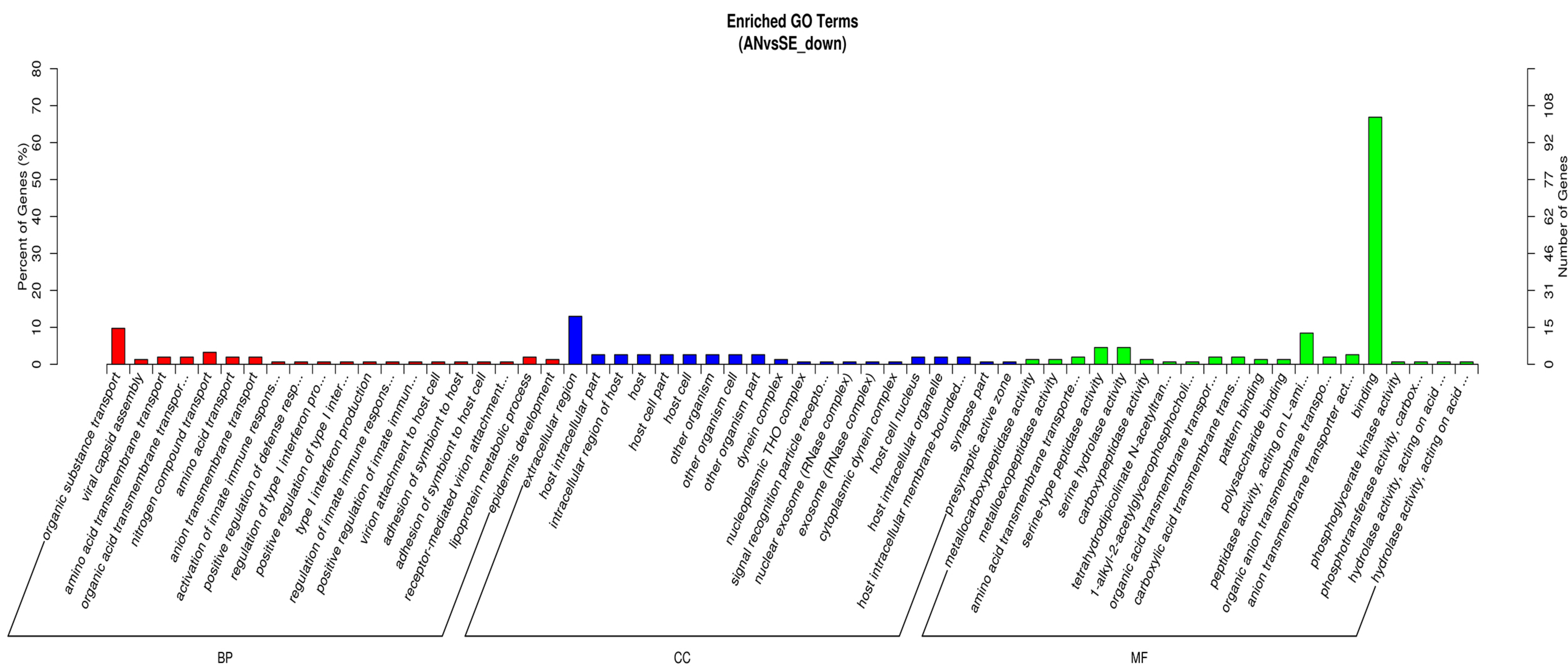

F

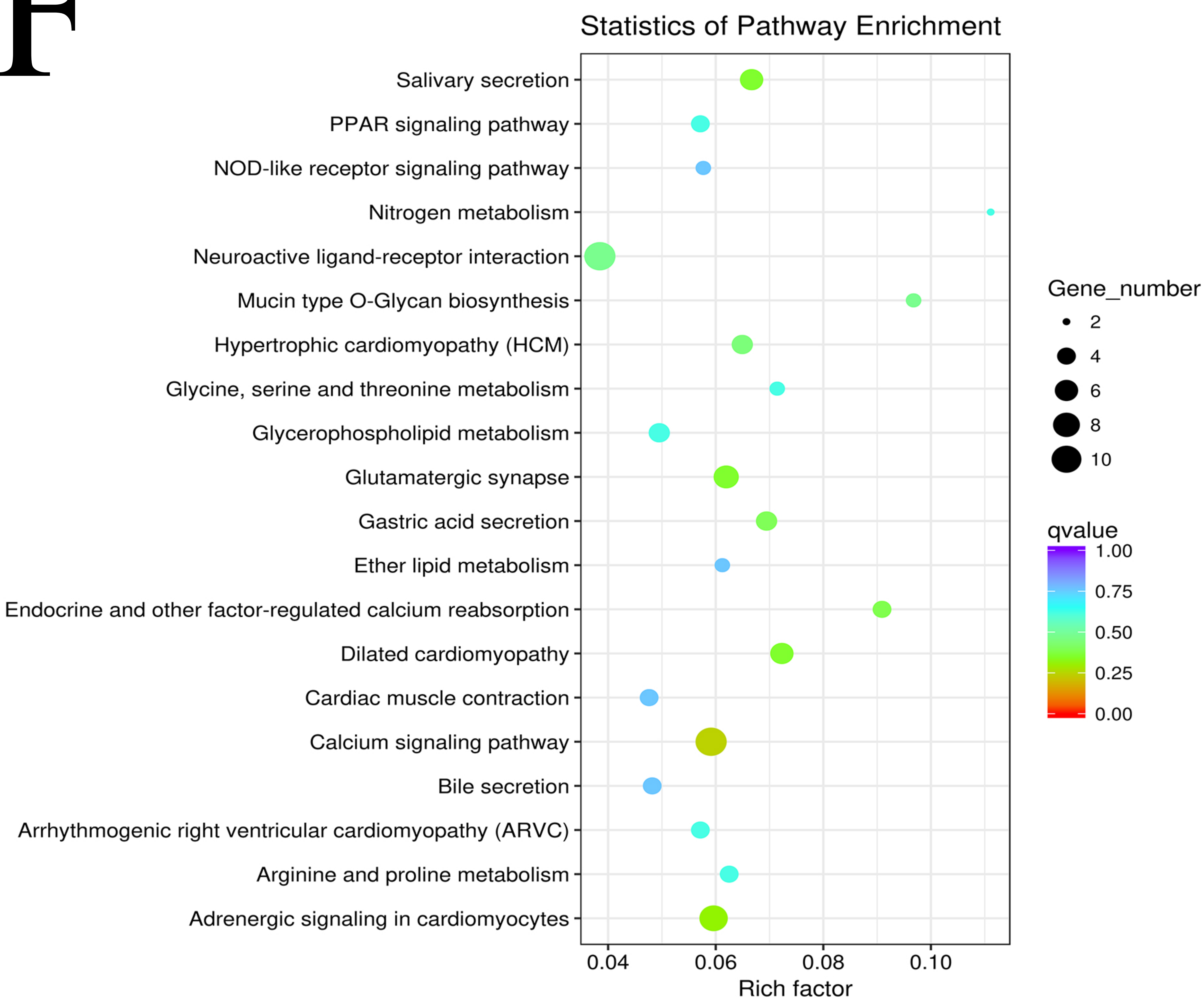

Supplement: Supplementary file 12 [file Data_Sheet_3.PDF]
